# Frozen Fermented Dairy Snacks with Probiotics and Blueberry Bagasse: Stability, Bioactivity, and Digestive Viability

**DOI:** 10.3390/microorganisms13010086

**Published:** 2025-01-04

**Authors:** Alejandra Hurtado-Romero, Andrea Zepeda-Hernández, Javier Cárdenas-Rangel, Ricardo Aguilar-Márquez, Luis Eduardo Garcia-Amezquita, Danay Carrillo-Nieves, Tomás García-Cayuela

**Affiliations:** 1 Tecnologico de Monterrey, Escuela de Ingeniería y Ciencias, Ave. General Ramón Corona 2514, Zapopan 45138, Mexico; 2 Tecnologico de Monterrey, Escuela de Ingeniería y Ciencias, Ave. Eugenio Garza Sada 2501, Monterrey 64849, Mexico

**Keywords:** frozen snack, dairy product, blueberry by-product, probiotic viability, functional food, fermented food

## Abstract

The demand for healthier snack options has driven innovation in frozen dairy products. This study developed and characterized novel frozen dairy snacks fermented with probiotics (*Lactobacillus acidophilus* LA5; *Lacticaseibacillus rhamnosus* GG, and *Streptococcus thermophilus* BIOTEC003) and containing 2% blueberry bagasse. Four formulations (LA5, LGG, LA5-BERRY, and LGG-BERRY) were analyzed for their nutritional, physicochemical, functional, and sensory properties. High protein content (>17% d.w.) and increased dietary fiber (5.77–5.88% d.w.) were observed in bagasse-containing formulations. Stable technological characteristics were maintained, with melting rates increasing slightly during storage. Probiotic viability remained high (>8.5 log CFU/mL) after freezing and storage at −20 °C for 30 days. Post-simulated digestion, probiotics retained >7.5 log CFU/mL, while blueberry bagasse formulations exhibited significantly higher phenolic content (7.62–8.74 mg/g d.w.) and antioxidant capacity, though anthocyanin content decreased (66–68%). Sensory evaluation by 100 panelists revealed high acceptance scores (>63%), with LGG-BERRY achieving the highest score (78%). These formulations demonstrate significant potential for incorporating probiotics and functional ingredients, providing an innovative solution for probiotic delivery and the sustainable utilization of fruit by-products in the food industry.

## 1. Introduction

The snack market is among the fastest-growing segments in the food industry, with an annual growth rate of 5.14%. The COVID-19 pandemic lockdowns, coupled with the global shift toward hybrid working environments, have accelerated this growth over recent years. This trend is expected to continue, with projections estimating the healthy snack market value to reach USD 152.2 billion by 2030, compared to USD 96.6 billion in 2023 [1]. In recent years, consumer preferences have shifted from traditional unhealthy snacks like chocolates, biscuits, and chips to healthier alternatives, including fruit-based products, dairy items, and functional snacks [2]. Current trends in the snack industry emphasize the development of products with high protein content, reduced carbohydrates, increased dietary fiber, and optimized fatty acid profiles. These innovations target health-conscious consumers, particularly students and working professionals, seeking convenient, functional, and ready-to-eat foods [3].

Dairy-based snacks stand out for their high-quality protein, bioavailable nutrients, and versatile formulations. These qualities make them suitable for both indulgent treats and health-focused products [3]. Frozen dairy desserts, such as ice cream and frozen yogurt, are especially popular due to their sensory appeal and nutritional benefits. Additionally, these products serve as effective carriers for functional ingredients, such as probiotics, dietary fibers, and antioxidants derived from fruit-based ingredients. This aligns with consumer demand for innovative, health-promoting snacks [4].

Probiotics, defined as live microorganisms that confer health benefits when consumed in adequate amounts [5], have traditionally been incorporated into dairy products like yogurt, ice cream, and fermented beverages. The compatibility of dairy matrices with probiotics facilitates high microbial viability during storage, even under frozen conditions. Low storage temperatures not only preserve probiotics but also mitigate their exposure to stress, allowing these microorganisms to remain viable over prolonged periods [6,7]. For instance, whey proteins in dairy matrices have been shown to improve the viability of *Lactobacillus* and *Bifidobacterium* strains by providing a protective effect during storage [6]. Additionally, dairy ice cream has been reported as an excellent carrier for probiotics, offering a stable environment that supports their long-term viability and effective delivery to consumers [7].

Fruit by-products have been widely studied for their potential to enhance probiotic viability and functionality in fermented dairy products. While fruits like mango, mixed berries, passion fruit, and banana have been added to fermented milk, their acidic pH can reduce probiotic populations during storage [8]. In frozen applications, cornelian cherry peels incorporated into probiotic ice cream improved vitamin C, polyphenol, and anthocyanin content while maintaining *Bifidobacterium lactis* viability during freezing [9]. Other by-products, such as tomato skin, grape stems, and pomegranate peel, have shown prebiotic effects, enhancing probiotic growth and survival, which could support their use in frozen formulations [10]. Furthermore, fruit-derived pulp, seeds, and extracts have improved the sensory, physicochemical, and antioxidant properties of dairy-based products, indicating their potential for developing functional frozen snacks [11,12].

Among these by-products, blueberry bagasse stands out due to its rich content of phenolic acids, flavonoids, anthocyanins, and dietary fiber. Derived from juice extraction, it primarily consists of seeds, peels, pulp, and stems. These components confer antimicrobial, antioxidant, and potential prebiotic properties, supporting the proliferation of beneficial microorganisms and promoting gut health [13]. It has been successfully incorporated into low-cost, ready-to-eat cereal-based snacks with extended shelf life. However, its fiber-rich phenolic compounds may reduce antioxidant bioavailability by trapping these compounds within the fiber matrix, and processing conditions, such as temperature, can impact its functional properties [14]. In dairy products, such as Petit Suisse cheese fermented with probiotics, blueberry bagasse has enhanced both the nutritional profile and antioxidant properties, highlighting its potential for functional and innovative food applications [15].

Although significant research has been conducted on probiotics in dairy matrices and the valorization of fruit by-products, studies investigating the combined effects of probiotics and blueberry bagasse in frozen fermented dairy snacks remain scarce. While the use of blueberry by-products to enhance dairy products is an emerging area of interest, the impact of blueberry bagasse on the stability and functionality of frozen dairy snacks, particularly in combination with probiotics during storage and after simulated gastrointestinal digestion, has not been thoroughly addressed in the literature.

**Figure 1:**
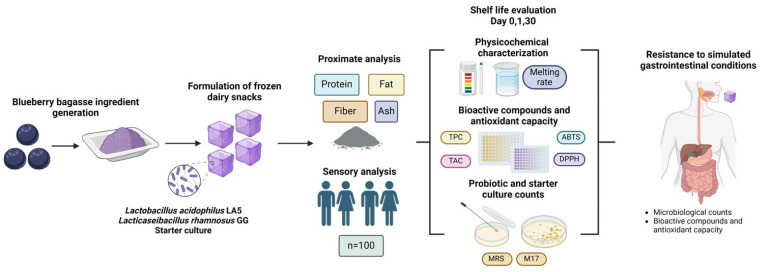
Experimental sequence for developing and evaluating the frozen dairy snacks fermented with probiotics and containing blueberry bagasse.

The aim of this study was to develop and characterize novel frozen dairy snacks fermented with probiotics and containing blueberry bagasse. Proximate composition and sensory analyses were conducted using the frozen product, while physicochemical properties, bioactive compounds, antioxidant capacity, and probiotic viability were evaluated during 30 days of storage at −20 °C. Additionally, bioactivity and probiotic survival were assessed after simulated gastrointestinal digestion to provide comprehensive insights into the product’s functionality and stability. The experimental sequence is presented in Figure 1.

## 2. Materials and Methods

### 2.1. Chemicals and Bacterial Strains

The commercial probiotics *Lactobacillus acidophilus* LA5 (Chr. Hansen) and *Lacticaseibacillus rhamnosus* GG (ATCC 7469) were grown using MRS (Man-Rogosa-Sharpe) broth and agar (BD Difco, Sparks, MD, USA). The potential probiotic *Streptococcus thermophilus* BIOTEC003, isolated from Mexican milk kefir [16], was grown using M17 broth and agar (BD Difco) supplemented with 0.5% of lactose (LM17). Ingredients and materials for the snack formulations and sensory analysis were purchased at a local supermarket in Guadalajara, Jalisco, Mexico. All reagents and chemicals used were of analytical grade. Digestive enzymes, Folin–Ciocalteu phenol reagent, ABTS (2,2-azino bis (3-ethylbenzo thiazoline 6 sulfonic acid)), DPPH (2,2-difenil-1-picrilhidrazil), and other analytical reagents were obtained from Sigma-Aldrich (St. Louis, MO, USA).

### 2.2. Preparation of the Blueberry Bagasse Ingredient

A lyophilized powder was obtained from blueberry bagasse, as described previously [15]. Briefly, second-quality blueberry fruits (*Vaccinium myrtillus* L., cv. Kirra) were harvested from Bloom Farms^®^, an orchard located in Amatitán, Jalisco, Mexico. The blueberries underwent a series of steps, including washing, draining, blending, and centrifugation at 3000× *g* for 15 min to separate the juice from the bagasse (comprising the residual pulp, peel, and seeds). The collected bagasse was lyophilized at −83 °C, 0.035 mbar, using a FreeZone 4.5 freeze dryer (Labconco, Kansas City, MO, USA). After lyophilization, the material was ground using an IKA A10 basic analytical grinder (IKA-Werke, Staufen im Breisgau, Germany). The particle size was further reduced by sieving through 105 µm mesh. The powdered ingredient was then stored at −20 °C until its utilization.

### 2.3. Preparation of Frozen Fermented Dairy Snacks

Probiotic strains *L. acidophilus* LA5 and *L. rhamnosus* GG were subcultured twice and grown anaerobically in MRS broth at 37 °C for 24 h. Similarly, *S. thermophilus* BIOTEC003, used as a starter strain, was subcultured twice and grown in L-M17 broth under the same conditions. After cultivation, all cultures were harvested by centrifugation (3000× *g*, 10 min, 4 °C), resuspended in sterile saline solution, inoculated into ultra-pasteurized skim milk (1% of fat; Sello Rojo, Guadalajara, Mexico), and incubated at 37 °C for 16 h. All cultures reached cell counts of 9.0–9.3 log colony-forming units (CFU)/mL after growth in milk.

Four formulations of frozen fermented dairy snacks were produced, as shown in Table 1. First, ultra-pasteurized skim milk (1% of fat; Sello Rojo, Mexico) was heated to 85 °C to dissolve powdered milk (Alpura, Cuautitlán Izcalli, Mexico), then cooled to 37 °C before inoculation. The base formulation involved inoculating *S. thermophilus* BIOTEC003 culture into the skim milk for all variants. Additionally, either *L. acidophilus* LA5 or *L. rhamnosus* GG was separately inoculated into the milk. Immediately after inoculation into the skim milk matrix, the cell counts were 7.1–7.4 log CFU/mL for *S. thermophilus* BIOTEC003 and 6.8–7.2 log CFU/mL for *L. acidophilus* LA5 and *L. rhamnosus* GG. All base formulations were fermented for 16 h at 37 °C. Then, the fermented milk was filtered (2 h, 4 °C) using cheesecloth, and sucralose was incorporated into each formulation. The differentiation between the formulations was the inclusion of blueberry bagasse powder in two variants, resulting in the LA5-BERRY and LGG-BERRY snacks. To ensure all formulations had the same appearance and flavor, especially for sensory tests, an artificial coloring (Badia Spices, Doral, FL, USA) and artificial flavoring (Deiman, Ciudad de México, Mexico) were added to the control formulations (LA5 and LGG) to simulate the color of the blueberry. All formulations were produced in triplicate, poured into silicone molds, and frozen at −20 °C. They were stored for 30 days and subsequently used for physicochemical characterization, microbiological viability, simulated gastrointestinal conditions, and sensory analysis. For proximate analysis and the characterization of bioactive compounds and antioxidant activity, the samples were lyophilized and ground under the same conditions described in Section 2.2.

### 2.4. Proximate Analysis

The proximate composition was determined as described in the official AOAC methods. Protein content was analyzed using the Kjeldahl method (AOAC method 920.152), while lipid content was measured using the Goldfisch method (AOAC method 960.39). Moisture content (total solids) was determined by drying the sample at 60 °C to a constant weight (AOAC method 920.151). Ash content was analyzed by heating the sample at 525 °C for 5 h (AOAC method 940.26). Additionally, total high molecular weight dietary fiber (HMWDF), as well as its soluble (SDF) and insoluble (IDF) fractions, were determined using the AOAC method 2011.25. Digestible carbohydrates were calculated by difference.

### 2.5. Physicochemical Characterization

Frozen snack samples were analyzed on the same day they were prepared (day 0), one day after freezing (day 1), and after 30 days of storage at −20 °C (day 30). The timing of the analyses depended on the specific technique being applied. The melting rate was measured according to Akalın et al. (2018) by placing 60 g of frozen sample on a 1 mm wire mesh positioned over a beaker at room temperature (23–24 °C). After 90 min, the weights of the frozen and melted fractions were recorded, and the melting rate was calculated as the weight of the drip divided by the time [17]. Viscosity, pH, and color were also evaluated at room temperature. Viscosity was measured using a CVP-8S viscometer (CScientific, Beijing, China) following the manufacturer’s instructions (torque between 40–50%). The pH was determined using a pH meter (Horiba LAQUAact-PH110-K, Kyoto, Japan). Color parameters (L*, a*, b*) were measured with a Minolta spectrophotometer (Minolta Co., Ltd., Kyoto, Japan) using the CIE system. Titratable acidity was determined according to the Mexican Official Standard 155-SCFI-2012 by titration with 1 mol/L NaOH, and results were expressed in g of lactic acid/L of fresh weight.

### 2.6. Determination of Total Bioactive Compounds and Antioxidant Capacity

#### 2.6.1. Methanolic Extracts Preparation

The extraction procedure was adapted from García-Cayuela et al. (2019) with modifications [18]. First, 0.5 g of freeze-dried samples were mixed with 5 mL of methanol/water (50:50, *v*/*v*) and subjected to ultrasonic bath treatment (2 min, 25 °C, 40 kHz). After centrifugation for 15 min (3000× *g*, 4 °C), the supernatant was collected, and the solid residue was re-extracted twice with 3 mL methanol/water (50:50, *v*/*v*), followed by a final extraction with 3 mL of 100% methanol. The combined supernatants were concentrated to a minimum volume using a rotary evaporator at 25 °C (IKA RV 10; IKA-Werke, Staufen im Breisgau, Germany) and subsequently resuspended in 5 mL of MilliQ water for spectrophotometric analysis.

#### 2.6.2. Total Phenolics and Monomeric Anthocyanins

The total phenolic content (TPC) and total monomeric anthocyanin content (TAC) were determined using the Folin–Ciocalteu assay and the pH-differential method, respectively, both adapted for a 96-well microplate format [19,20]. Measurements were conducted with a microplate reader (Varioskan Lux, Thermo Fisher Scientific, Waltham, MA, USA). TPC was quantified by measuring absorbance at 765 nm, with gallic acid as the standard. Results were expressed as mg of gallic acid equivalents (GAE) per g of dry weight (d.w.). TAC was assessed by measuring absorbance at 510 and 700 nm and expressed as µg of cyanidin-3-glucoside equivalents (C3G) per g of d.w.

#### 2.6.3. Antioxidant Activity

The antioxidant activity of the extracts was determined using the ABTS and DPPH assays, following the method described by Martín-Gomez et al. (2020) [8]. For the ABTS assay, absorbance was measured at 734 nm, while for the DPPH assay, it was measured at 515 nm. The results for scavenging activity were expressed as mg of Trolox equivalents per g of d.w.

### 2.7. Microbiological Viability

The viability of probiotics and the starter culture in the snack formulations was assessed using the plate count method. Samples were taken on days 1 and 30 of cold storage, and after in vitro digestion. The samples were then serially diluted and plated onto MRS or L-M17 agar. Plates were incubated at 37 °C for 48 h. Results were expressed as CFU/mL to quantify viable colonies.

### 2.8. Simulated Gastrointestinal Conditions

The in vitro simulated gastrointestinal digestion of frozen snacks was conducted following the INFOGEST protocol [21] with modifications. Samples were mixed 1:1 (*v*/*v*) with simulated digestion fluids, including simulated salivary fluid (SSF), simulated gastric fluid (SGF), and simulated intestinal fluid (SIF). These fluids were composed of KCL (46.7 g/L), KH_2_PO_4_ (68 g/L), NaHCO_3_ (84 g/L), NaCl (120 g/L), and MgCl_2_(H_2_O)_6_ (30 g/L). In the oral phase, the sample was diluted 1:1 with SSF, containing 75 mg of amylase, adjusted to pH 7, and incubated for 2 min at 37 °C and 100 rpm. In the gastric phase, the sample was further diluted 1:1 with SGF, containing 160 mg of pepsin, adjusted to pH 3, and incubated for 2 h under the same conditions. Finally, in the intestinal phase, the sample was diluted 1:1 with SIF, bile salts (0.1 mol/L), and 40 mg of pancreatic enzymes, adjusted to pH 7, and incubated for another 2 h. Following the intestinal phase, 1 mL of sample was collected immediately for microbial counts. The final digested samples were lyophilized and stored at −80 °C for bioactive compound and antioxidant characterization.

### 2.9. Sensory Analysis

The sensory attributes of the frozen snacks were evaluated by 100 untrained panelists recruited based on their status as avid consumers of dairy products. The panelists, aged between 17 and 40 years (50% female and 50% male), participated in the evaluation conducted at the Food and Biotech Laboratory of Tecnológico de Monterrey. The sensory analysis took place under controlled conditions, including artificial illumination, air circulation, and a temperature of 23 °C. Panelists were informed about the protocols and samples, and written consent was obtained from all participants. The research protocol was approved by the Institutional Research Ethics Committee (CIEI) of Tecnológico de Monterrey (CA-EIC-2406-02). Frozen snack formulations were served at a temperature of −4 °C, with random codes assigned to each sample. Each panelist received 20 g of the samples in disposable transparent plastic cups and evaluated them using a 9-point hedonic scale, where 1 corresponded to “I dislike it very much” and 9 to “I like it very much” [22,23]. To ensure unbiased evaluation, water was provided to rinse the palate between samples. The sensory attributes assessed included taste, color, texture, melting behavior, and overall liking [24]. The acceptance index for each attribute was calculated as a percentage by dividing the mean score by the highest possible score [25].

### 2.10. Statistical Analysis

The statistical analysis was performed using data from two biological replicates, each comprising at least three technical measurements. Results are presented as means with standard deviations. Analysis of variance (ANOVA) was used to identify significant differences among groups, followed by Tukey’s multiple comparison test for post-hoc analysis. Additionally, paired comparisons were conducted using the T-student test where applicable. A significance level of 0.05 (*p*-value) was applied to all tests. All analyses were carried out using Minitab Software 21.4 (Minitab, LLC, State College, PA, USA)

## 3. Results

### 3.1. Proximate Composition

The proximate composition of the frozen fermented dairy snacks is shown in Table 2. On a dry weight basis, no significant differences were observed in moisture, ash, carbohydrate, fat, or protein content among the four formulations, except for dietary fiber. Protein content ranged from 17.25% to 20.39% d.w., classifying the snack as a high-protein product. This aligns with its suitability as a functional and nutrient-rich frozen dairy snack. The most notable differences were observed in dietary fiber content, as expected from the addition of blueberry bagasse. The LA5-BERRY and LGG-BERRY formulations had significantly higher high-molecular-weight dietary fiber levels (5.77–5.88 g/100 g d.w.) compared to their controls. Insoluble dietary fiber content was also significantly higher in blueberry bagasse formulations, with values of 4.13 and 4.11 g/100 g d.w., respectively, while soluble dietary fiber content showed no differences among formulations. The higher dietary fiber content in blueberry bagasse formulations aligns with current trends in health-oriented snack development, emphasizing fiber enrichment as a key nutritional attribute.

### 3.2. Physicochemical Parameters

**Figure 2:**
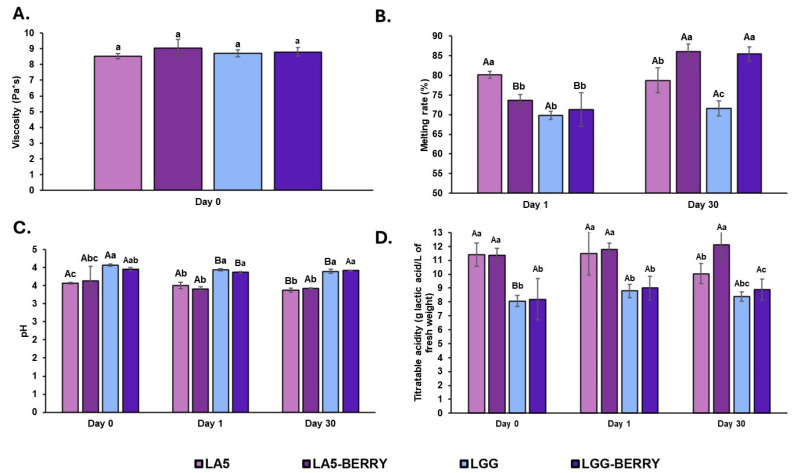
Physicochemical parameters (**A**), viscosity; (**B**), melting rate; (**C**), pH; and (**D**), titratable acidity) of frozen fermented dairy snack formulations immediately on the same day they were prepared (day 0), one day after freezing (day 1), and after 30 days of storage at −20 °C (day 30). Capital letters indicate significant differences (*p* < 0.05) among days of storage, and lower-case letters indicate significant differences among formulations. The error bars indicate the standard deviation. Frozen snack formulations are described in Table 1.

Viscosity was measured on day 0, after mixing the ingredients with fermented milk but before freezing (Figure 2A). No significant differences in viscosity were observed among the formulations, with values ranging from 8.53 to 9.03 Pa·s. The observed viscosity stability across formulations indicates that the inclusion of 2% blueberry bagasse does not compromise the flow properties of the dairy matrix, suggesting compatibility with processing and storage conditions.

The melting rate was evaluated on day 1 (24 h after freezing) and after 30 days of storage at −20 °C (Figure 2B). On day 1, LA5 exhibited a significantly higher melting rate (80.21%) compared to the other formulations (69.82–73.70%). By day 30, significant differences were observed in melting rates, with LA5-BERRY and LGG-BERRY showing the highest values (86.02% and 85.41%, respectively). In contrast, LGG had the lowest melting rate after 30 days (71.58%), and LGG-BERRY displayed the greatest increase during storage, rising from 71.33% to 85.41%.

The pH and titratable acidity were measured on day 0, day 1, and day 30. On day 0, LA5 had the lowest pH (3.57), which was significantly different from LGG, exhibiting the highest pH (4.07). On day 1, slight decreases in pH were observed in LGG and LGG-BERRY (3.94 and 3.87, respectively). After 30 days, significant pH reductions were recorded in LA5 and LGG (3.57 to 3.37 for LA5 and 4.07 to 3.89 for LGG), whereas the pH of samples containing blueberry bagasse remained stable (Figure 2C).

For titratable acidity (Figure 2D), LA5 and LA5-BERRY displayed significantly higher values on day 0 (11.42 and 11.38 g of lactic acid/L, respectively) compared to LGG and LGG-BERRY (8.07 and 8.19 of lactic acid, respectively). On day 1, no significant differences were observed between formulations or compared to day 0. By day 30, only LA5 showed a slight decrease in acidity (10.03 g lactic acid/L), while the other formulations maintained their levels.

Color parameters (L*, a*, b*) were assessed on days 0, 1, and 30 (Table 3). Significant differences (*p* < 0.05) were observed among formulations and storage times. On day 0, LA5 and LGG had similar lightness values, which were significantly higher than LA5-BERRY and LGG-BERRY. Over time, all formulations showed a decrease in L*, especially LA5-BERRY. Formulations with blueberry bagasse displayed significantly higher a* values compared to their controls, indicating increased redness. This trend persisted across storage days, though slight variations were observed. In contrast, LA5-BERRY and LGG-BERRY had less negative b* values compared to control formulations, reflecting a reduction in the blue hue. Over time, all formulations showed variations in b*, with the magnitude of change depending on the presence of blueberry bagasse. It is important to note that the control formulations (LA5 and LGG) contained an artificial blueberry coloring to simulate the blueberry’s color. This difference in ingredient composition may have contributed to the observed disparities in color parameters.

**Table 3:** CIELAB color parameters ^1^ of frozen fermented dairy snack formulations.

		Formulations ^2^			
		LA5	LA5-BERRY	LGG	LGG-BERRY
Day 0	L*	73.44 ± 0.05 ^Aa^	60.31 ± 0.07 ^Ab^	73.39 ± 0.10 ^Aa^	60.68 ± 0.21 ^Ab^
	a*	11.15 ± 0.02 ^Cb^	12.24 ± 0.04 ^Ca^	10.75 ± 0.09 ^Cb^	10.33 ± 0.09 ^Cc^
	b*	−5.74 ± 0.02 ^ABb^	−1.06 ± 0.03 ^Aa^	−6.05 ± 0.06 ^Ab^	−0.98 ± 0.05 ^Aa^
Day 1	L*	62.10 ± 0.69 ^Ba^	50.24 ± 0.33 ^Bb^	58.35 ± 0.53 ^Ba^	48.53 ± 0.33 ^Bb^
	a*	13.75 ± 0.17 ^Bb^	15.69 ± 0.20 ^Aa^	14.49 ± 0.08 ^Bb^	12.68 ± 0.01 ^Ac^
	b*	−6.48 ± 0.11 ^Bb^	−2.03 ± 0.03 ^Ca^	−5.98 ± 0.07 ^Ab^	−1.84 ± 0.01 ^Ba^
Day 30	L*	58.77 ± 0.17 ^Ca^	40.55 ± 0.09 ^Cc^	57.80 ± 0.14 ^Ca^	47.35 ± 0.05 ^Cb^
	a*	14.77 ± 0.42 ^Aa^	15.41 ± 0.06 ^Ba^	14.74 ± 0.06 ^Aa^	10.89 ± 0.03 ^Bb^
	b*	−4.05 ± 0.07 ^Ab^	−1.48 ± 0.04 ^Ba^	−5.98 ± 0.04 ^Ab^	−0.73 ± 0.02 ^ABa^

^1^ Means ± standard deviation. Capital letters in the same column indicate significant differences (*p* < 0.05) among days of storage. Lower case letters in each row indicate significant differences (*p* < 0.05) among formulations; ^2^ Frozen snack formulations are described in Table 1.

### 3.3. Evaluation of Total Bioactive Compounds and Antioxidant Capacity Before and After Digestion

Table 4 presents the total phenolic content (TPC), total anthocyanin content (TAC), and antioxidant activity of frozen fermented dairy snack formulations stored for 30 days, both before and after in vitro digestion. Significant differences were observed across all frozen dairy snack samples following simulated gastrointestinal digestion.

Before digestion, the formulations containing blueberry bagasse had the highest TPC values, ranging from 1.74 to 1.85 mg GAE/g d.w. They also exhibited similar TAC values (42.92 and 46.57 µg C3G/g d.w.), with no significant differences between the two formulations. Additionally, these samples demonstrated superior antioxidant capacity in the ABTS assay. In contrast, antioxidant capacities measured by the DPPH assay were comparable across all formulations, regardless of the presence of blueberry bagasse. Our previous study reported that the antioxidant capacity of blueberry bagasse by ABTS was 23.84 mg/g d.w. [15]. The values observed for the frozen snack formulations with 2% blueberry bagasse are comparable with these findings, considering the proportion of the ingredient used and the potential interactions within the dairy matrix that may influence the results [15].

After digestion, TAC significantly decreased in the formulations containing blueberry bagasse, while TPC showed a marked increase, particularly in the LA5-BERRY sample (8.74 mg GAE/g d.w.), followed by LGG-BERRY (7.62 mg GAE/g d.w.). Antioxidant capacity improved across all samples post-digestion, as evidenced by both the ABTS and DPPH assays. Notably, blueberry bagasse-containing formulations demonstrated significantly higher antioxidant capacity in the ABTS assay compared to their controls.

### 3.4. Microbiological Viability During Storage at −20 °C

**Figure 3:**
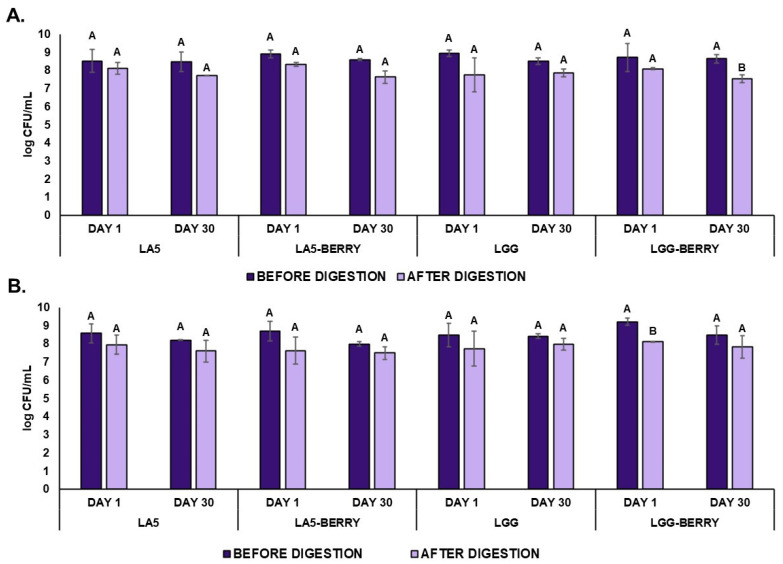
Viability (log CFU/mL) of probiotics *Lactobacillus acidophilus* LA5 and *Lacticaseibacillus rhamnosus* GG in MRS medium (**A**) and *Streptococcus thermophilus* BIOTEC003 in L-M17 medium (**B**) within frozen fermented dairy snack formulations before and after in vitro digestion (intestinal phase) on day 1 and day 30 during storage at −20 °C. Error bars indicate the standard deviation. Capital letters represent significant differences before and after in vitro digestion within each formulation. No significant differences were observed between storage days (*p* < 0.05). Frozen snack formulations are described in Table 1. CFU, Colony Forming Units.

The viability of probiotics *L. acidophilus* LA5, *L. rhamnosus* GG, and *S. thermophilus* BIOTEC003 in frozen snack formulations was monitored during the freezing stage, as shown in Figure 3. On day 1, probiotic counts ranged between 8.5 and 8.9 log CFU/mL, with no significant differences observed among formulations. Similarly, *S. thermophilus* BIOTEC003 counts exceeded 8.5 log CFU/mL, with the LGG-BERRY formulation reaching 9.2 log CFU/mL, comparable to other formulations. Both blueberry bagasse-containing formulations and control formulations demonstrated similar maintaining levels close to the initial counts of 8.9–9.4 log CFU/mL for the probiotics and 8.7–9.2 log CFU/mL for *S. thermophilus* BIOTEC003 recorded before freezing. These results indicate that the freezing process had minimal impact on the viability of probiotics and starter cultures across all formulations.

After 30 days of storage, probiotic viability remained within a range of 8.5–8.6 log CFU/mL, with no significant differences observed between formulations. Specifically, the LGG-BERRY formulation maintained a viability of 8.6 log CFU/mL, showing no significant differences over the storage period. For S. thermophilus BIOTEC003, viability ranged from 8.0 to 8.5 log CFU/mL, with the LGG-BERRY formulation retaining a count of 8.5 log CFU/mL. Similar to the probiotics, no significant differences were reported between formulations in starter culture viability, confirming stability throughout the freezing storage.

### 3.5. Microbiological Resistance to Simulated Gastrointestinal Conditions

To evaluate the survival of probiotics and *S. thermophilus* BIOTEC003 to simulated gastrointestinal conditions, samples were taken at the intestinal stage of digestion on day 1 and day 30. The results are also shown in Figure 3. There were no significant differences between day 1 and day 30 in the culture counts of both probiotics and *S. thermophilus* BIOTEC003 of all the formulations. Overall, after the intestinal phase on day 1, a slight decrease in probiotic counts was observed, ranging from 0.4 to 1.2 log CFU/mL, although no significant differences were detected between formulations. LA5 and LA5-BERRY showed strong resistance to simulated gastrointestinal conditions, maintaining final concentrations of 8.1 and 8.3 log CFU/mL, respectively, while LGG and LGG-BERRY reached 7.7 and 8.1 log CFU/mL, respectively. These results highlight the robust resistance of probiotics to in vitro digestion, with no significant differences noted between digestion phases for any of the formulations. For *S. thermophilus* BIOTEC003, no significant reductions in viability were observed after the intestinal phase, except for LGG-BERRY on day 1, which showed a slight but significant reduction of 1.1 log CFU/mL after digestion. By day 30, probiotic and starter culture counts in the intestinal phase ranged from 7.5 to 7.8 log CFU/mL, depending on the formulation. A significant difference was noted only for the LGG-BERRY formulation, which showed a reduction in intestinal phase viability from 8.6 log CFU/mL (initial phase) to 7.6 log CFU/mL. These results confirm the resilience of probiotics and starter cultures in frozen matrices, underscoring their suitability for functional frozen snack applications.

### 3.6. Sensory Evaluation

**Figure 4:**
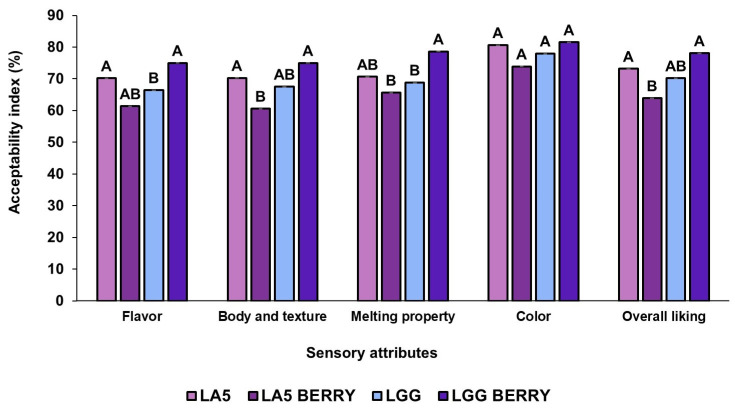
Sensory properties of frozen fermented dairy snack formulations were evaluated based on the acceptability index using a 9-point hedonic scale. Capital letters indicate significant differences (*p* < 0.05) among formulations. The error bars indicate the standard deviation. Frozen snack formulations are described in Table 1.

The sensory attributes of the frozen formulations, such as flavor, texture, melting behavior, color, and overall liking, were evaluated by 100 panelists (Figure 4). All formulations were well-accepted, with overall liking scores above 63%. When comparing blueberry bagasse formulations to their controls, LA5-BERRY scored lower in most attributes than LA5, while LGG-BERRY consistently outperformed LGG. Among the blueberry bagasse formulations, LA5-BERRY had the lowest overall liking (63.92%) and received lower scores for texture (60.62%) and melting behavior (65.68%). In contrast, LGG-BERRY achieved the highest overall liking (78.11%) and stood out in texture (75.03%) and melting behavior (78.66%). The blueberry bagasse contributed to distinct flavor and texture profiles, which were preferred in LGG-BERRY but rated lower in LA5-BERRY, reflecting strain-specific interactions with the matrix. Artificial coloring and flavoring were used in the control formulations (LA5 and LGG) to simulate blueberry attributes. These additives may have influenced sensory evaluations, particularly for flavor and color. However, no significant differences were observed in color preference between formulations.

## 4. Discussion

The increasing demand for healthier and more appealing foods is driving the dairy industry to develop innovative products that combine nutritional benefits with convenience, such as probiotic and synbiotic options in ready-to-eat formats. Frozen snacks, known for their health-promoting properties, high consumer acceptance, and broad appeal, are an excellent platform for such innovations [26]. This study aimed to develop novel frozen dairy snacks fermented with probiotics and containing blueberry bagasse, aligning with emerging trends toward protein- and fiber-enriched and reduced-fat formulations. The inclusion of 2% bagasse was selected as an optimal concentration to balance functionality, consumer acceptance, and health-promoting attributes in dairy products. Previous studies support the effectiveness of incorporating 2% of blueberry bagasse or fruit-derived fibers into dairy matrices such as ice cream and Petit Suisse [15,17].

The proximate analysis showed that formulations containing blueberry bagasse had a high protein content (17.3–18.1% d.w.) with lower levels of fat (9.5% d.w.). These findings are consistent with trends in snack development reported in the literature [2]. Equivalent amounts of these nutrients have been reported for other dairy-based snacks, including frozen yogurt formulations enriched with purple sweet potato [27] and Petit Suisse formulations [13]. The addition of 2% blueberry bagasse significantly increased dietary fiber content, with LA5-BERRY and LGG-BERRY showing 5.77% (d.w.) and 5.88% (d.w.), respectively, compared to their controls (1.92–2.05% d.w.). This increase is primarily attributed to the high levels of insoluble dietary fiber present in blueberry bagasse [13]. These results align with studies on fruit by-products like orange peel powder, where the incorporation of 2.5% orange peel powder in frozen yogurt increased crude fiber content to 4.37% (d.w.) compared to 1.29% (d.w.) in the control [28]. Furthermore, the addition of 2% blueberry bagasse doubled the fiber content in Petit Suisse formulations [15]. This demonstrates the valuable contribution of blueberry bagasse as a source of dietary fiber in dairy-based snacks.

Consumer preference for ice cream and frozen desserts is strongly influenced by their chemical composition and sensory attributes, such as texture, consistency, melting resistance, taste, and other physicochemical characteristics [29]. Despite reports that adding sugars, fruit pulps, or fibers can impact viscosity [30], the inclusion of 2% blueberry bagasse did not alter viscosity in our formulations. This suggests that the blueberry bagasse is compatible with frozen dairy products, likely due to its high insoluble dietary fiber content and its interaction with the dairy matrix. Previous studies have shown that viscosity changes in fiber-enriched probiotic ice cream depend on the fiber’s composition and water-binding properties. Fibers with a balanced ratio of soluble to insoluble fractions, like apple or orange fibers, increase viscosity due to soluble components such as pectin, while fibers with predominantly insoluble fractions, such as oat fiber, have minimal impact due to their grainy morphology [17]. Similarly, the high insoluble fiber content and particle morphology of blueberry bagasse likely explain the absence of viscosity changes in this study, with the frozen dairy matrix potentially stabilizing the formulation without significant thickening.

Viscosity plays a significant role in the melting behavior of ice cream and frozen desserts. Higher viscosity, often achieved by adding stabilizers, can enhance stability, reduce ice crystal size, and slow melting rates [31]. In this study, after 30 days at −20 °C, formulations with blueberry bagasse (LA5-BERRY and LGG-BERRY) showed the highest melting rates. This increase in melting rate over time may be explained by structural changes in the frozen dairy matrix during storage, likely influenced by the fiber content and water-binding properties of the blueberry bagasse [32]. Bilbao-Sainz et al. (2019) reported that the use of blueberry powder in frozen desserts did not significantly improve melting resistance compared to control samples. This behavior was attributed to the fiber content and the formation of milk protein-blueberry pectin complexes, which are unstable and prone to sedimentation [33]. Moreover, characteristics such as meltdown time, water-binding capacity, and the first dripping point of ice cream are intrinsically affected by fiber content, fat destabilization, and the overall structural arrangement of the food matrix [34]. These findings reinforce the critical influence of formulation components on the rheological and structural properties of frozen desserts.

The pH for the formulations with *L. acidophilus* LA5 was significantly lower than those with *L. rhamnosus* GG, reflecting higher acidity levels in the LA5 formulations. Lower pH values in dairy products are typically associated with higher titratable acidity, which can result from organic acids present in the fruit or produced during fermentation [35]. In this study, the addition of blueberry bagasse had minimal impact on pH, suggesting that changes in pH and acidity were primarily driven by the activity of the probiotic microorganisms. Additionally, freeze concentration processes in frozen desserts can further decrease pH and increase titratable acidity due to higher total solids and protein concentrations, which enhance the buffering capacity of the matrix [36]. These factors collectively influence the acidity profile of the final product.

Color parameters also underwent significant changes during storage, particularly in lightness (L*) and redness (a*), both of which are critical to consumer acceptance of frozen products. The decrease in L* values across all samples after 30 days suggests physical degradation or structural changes in the product due to storage conditions. These findings are consistent with those of Shamshad et al. (2023), who observed decreases in L* values and increases in a* values in ice cream enriched with microencapsulated anthocyanins from black carrots during storage [37]. Similar changes in color parameters were observed in the present study, even though the control formulations contained artificial colorants, emphasizing the impact of storage conditions on the visual appeal of the products.

With respect to bioactive compounds, the total anthocyanin content (TAC) was notably higher in formulations with blueberry bagasse compared to other frozen desserts reported in the literature. For example, TAC in soy milk-tofu-based frozen desserts with 7.8% puree or blueberry juice ranged from 4 to 5.6 µg/g [38], while in the current study, frozen snacks with blueberry bagasse exhibited 42.93 to 46.57 µg/g. After simulated gastrointestinal digestion, a significant increase in total phenolic content (TPC) was observed in all frozen dairy snack samples, while TAC significantly decreased in samples with blueberry bagasse, consistent with the known instability of anthocyanins during digestion [39]. These findings align with studies such as Du and Myracle (2018), which reported an increase of up to 96.9% in bioaccessible polyphenols during digestion in aronia kefir, even though bioaccessible anthocyanins were significantly reduced [39]. The increase in TPC can be attributed to the degradation of anthocyanins during digestion and the release of bound phenolics from the food matrix. Anthocyanin hydrolysis, driven by the breakdown of glycosidic bonds, produces smaller phenolic compounds that contribute to the rise in TPC [40]. Gastrointestinal conditions, such as acidic pH, enzyme activity, and interactions with dietary components, facilitate anthocyanin degradation and the release of these bound phenolics [41,42]. Additionally, the dairy matrix and digestion process may influence the analytical results, as reducing substances like peptides or amino acids could interact with the Folin–Ciocalteu assay. These observations underscore the complexity of phenolic transformations during digestion and highlight the critical role of the food matrix in influencing the bioaccessibility properties of the final product.

Frozen snacks enriched with blueberry bagasse demonstrated higher antioxidant capacity in the ABTS assay, which further increased after simulated gastrointestinal conditions. This aligns with literature reporting an overall increase in radical scavenging capacity during digestion, such as in yogurt matrices incorporating strawberries, where antioxidant capacity increased by 480% during the intestinal digestion phase [43]. Similarly, antioxidant capacity in the DPPH assay also increased after digestion, although no significant differences were observed among samples. The variability in antioxidant activity during different digestion phases is often attributed to pH fluctuations throughout the gastrointestinal process. These changes can alter the structure of phenolic compounds, thereby affecting their antioxidant potential [44]. The current results align with previous findings, where fermentation of blueberry bagasse with *L. plantarum* and *L. acidophilus* was shown to enhance both ABTS and DPPH radical scavenging activity. This increase was attributed to the metabolism of polyphenolic compounds, resulting in greater antioxidant activity and bioaccessibility in fermented blueberry pomace [45].

Regarding the functional viability of probiotics and the starter strain, *S. thermophilus* BIOTEC003, all formulations achieved a high initial concentration (>8.5 log CFU/mL). Remarkably, high viability was maintained during 30 days of storage at −20 °C and after in vitro digestion, with concentrations of at least 7.5 log CFU/mL in the intestinal phase. These results align with those reported by Şentürk et al. (2024), who evaluated the impact of blueberries on the viability of *Lactobacillus acidophilus* DSM 20079 in ice cream. Their study demonstrated that formulations preserved probiotic viability throughout a 60-day storage period, with counts ranging from 8.4 to 8.8 log CFU/g [46]. Similarly, *L. rhamnosus* GG in a passion fruit and whey protein beverage maintained populations >7.7 log CFU/mL during 28 days of storage at 5 °C and showed no significant reduction after simulated digestion [47]. Additionally, *L. acidophilus* LA5 in riceberry milk ice cream maintained populations above 5 log CFU/g in the intestinal phase after 60 days of frozen storage at −25 °C, especially when inulin was included to enhance stability during digestion [48]. Beyond ingredient selection and formulation, alternative strategies could be employed to enhance probiotic survival and extend product shelf life. For instance, the immobilization of *Pediococcus acidilactici* ORE5 cells on pistachio nuts in Katiki Domokou-type cheese sustained counts higher than 8.5 log CFU/g during 7 days of storage while also effectively controlling the presence of spoilage bacteria [49].

Our findings indicate that the incorporation of blueberry ingredients in frozen formulations does not negatively affect probiotic survivability. In this respect, Ahmad et al. (2020) reported that the addition of apple peel extracts provided a protective effect on the viability of *Lactobacillus acidophilus* and *Bifidobacterium lactis* in yogurt ice cream stored at −20 °C for 90 days. Viable counts of *L. acidophilus* in their samples ranged between 7.4 and 10.4 log CFU/g, with the yogurt ice cream fortified with 5% apple peel extract showing the highest probiotic counts [11]. This protective effect was attributed to the polyphenols present in the apple peel, which were suggested to act as prebiotics.

In our study, while no significant positive or negative effect of blueberry bagasse on the viability of microorganisms was observed, the potential interactions between blueberry phenolics and probiotics warrant further attention. Fermented products containing blueberry phenolics have been shown to enhance probiotic survival rates by exerting protective effects. Probiotics can metabolize phenolic compounds, releasing bound phenolics from the food matrix or transforming them into bioactive metabolites with higher bioavailability [44,50]. At the 2% concentration used in this study, blueberry bagasse did not inhibit probiotics, as evidenced by the comparable viability observed in formulations with and without blueberry bagasse. This indicates a balance in the interaction between phenolics and probiotics within the frozen dairy matrix. Additionally, the overall composition of the dairy matrix, including components such as proteins and residual lactose, may have played a stabilizing role, mitigating potential inhibitory effects [6,51]. These findings highlight the importance of factors such as phenolic concentration, structure, and matrix interactions in determining the overall impact of phenolics on probiotic viability and functionality.

Finally, sensory analysis results highlight the significant impact of incorporating fruit-based components into frozen fermented dairy snacks on sensory attributes. The positive overall liking of LGG-BERRY may be attributed to a potential synergistic interaction between *L. rhamnosus* GG and blueberry bagasse, which could enhance flavor, texture, and melting properties. Flavor remains a primary driver of consumer preference in ice cream products, even though texture and appearance also play important roles [52]. The addition of berry fruits, particularly in puree form, is generally well-accepted in ice cream formulations due to their contribution to flavor and color [53]. Similar results have been reported in studies such as those by Nascimento et al. (2018), where the inclusion of 2% grape by-products in ice cream yielded high sensory ratings across attributes like aroma, flavor, texture, and overall quality [54]. On the other hand, while artificial additives in the control formulations allowed for standardized sensory comparisons, they represent a limitation due to their lack of complexity and bioactivity compared to natural ingredients, potentially affecting flavor and color. This highlights the importance of selecting natural, bioactive-rich ingredients to meet consumer expectations for health-oriented products. Future studies should explore replacing artificial additives with natural alternatives to enhance both the authenticity and functional value of the formulations. The inclusion of blueberry bagasse not only optimizes sensory quality but also aligns with consumer preferences for functional and naturally derived frozen desserts.

Overall, this study establishes a framework for developing innovative probiotic frozen dairy snacks with fruit by-products like blueberry bagasse. Technologically, it demonstrates the feasibility of incorporating bioactive-rich ingredients with probiotic fermentation to create functional foods with balanced nutritional and sensory attributes. Economically, the valorization of blueberry bagasse offers a sustainable approach to reducing waste and adding value to the fruit industry. Scaling these formulations to industrial production will require optimizing production parameters, ensuring a consistent supply of raw materials, and implementing reliable quality control systems to facilitate the transition from laboratory-scale innovation to commercially viable products.

## 5. Conclusions

This study demonstrated that incorporating blueberry bagasse into probiotic frozen dairy snacks enhances their nutritional and functional profiles without compromising probiotic viability or sensory acceptance. Both LA5-BERRY and LGG-BERRY formulations emerged as high-fiber, antioxidant-rich snacks, with LGG-BERRY achieving superior sensory ratings. The survival of probiotics throughout freezing, storage, and simulated gastrointestinal digestion underscores the potential health benefits of these snacks as effective carriers for probiotics. These findings highlight the value of integrating sustainable ingredients, such as fruit by-products, into fermented dairy matrices. Future research should focus on extending shelf life, optimizing large-scale production processes, and validating health benefits through in vivo models and clinical trials.

## Figures and Tables

**Table 1:** Frozen fermented dairy snack formulations.

	Formulations			
	LA5	LA5-BERRY	LGG	LGG-BERRY
Microorganisms (%) ^1^				
*Streptococcus thermophilus* BIOTEC003	2	2	2	2
*Lactobacillus acidophilus* LA5	1	1	-	-
*Lactocaseibacillus rhamnosus* GG	-	-	1	1
Ingredients (%) ^2^				
Powdered milk	2.5	2.5	2.5	2.5
Sucralose	1	1	1	1
Blueberry bagasse powder	-	2	-	2
Artificial blueberry coloring	0.3	-	0.3	-
Artificial blueberry flavoring	0.3	-	0.3	-

^1^ mL of overnight culture/100 mL of milk. Before inoculation, all overnight cultures reached cell counts of 9.0–9.3 log colony-forming units/mL; ^2^ g of ingredient/100 g of fermented milk.

**Table 2:** Approximate composition of the frozen fermented dairy snack formulations.

Parameter ^1^	Formulations ^2^			
	LA5	LA5-BERRY	LGG	LGG-BERRY
Moisture (g/100 g fresh weight)	69.02 ± 2.28 ^a^	68.18 ± 7.62 ^a^	68.80 ± 2.42 ^a^	68.38 ± 1.45 ^a^
Protein	19.34 ± 2.33 ^a^	18.14 ± 0.27 ^a^	20.39 ± 0.82 ^a^	17.25 ± 0.87 ^a^
Ash	4.88 ± 1.19 ^a^	3.44 ± 0.60 ^a^	4.79 ± 1.32 ^a^	3.52 ± 0.30 ^a^
Fat	8.59 ± 0.70 ^a^	9.50 ± 0.68 ^a^	8.13 ± 0.60 ^a^	9.47 ± 1.08 ^a^
Digestible carbohydrates	65.14 ± 2.59 ^a^	63.15 ± 0.41 ^a^	64.77 ± 4.38 ^a^	63.88 ± 1.46 ^a^
HMWDF	2.05 ± 0.01 ^b^	5.77 ± 0.02 ^a^	1.92 ± 0.45 ^b^	5.88 ± 0.01 ^a^
HMWSDF	1.44 ± 0.03 ^a^	1.64 ± 0.00 ^a^	1.12 ± 0.45 ^a^	1.77 ± 0.22 ^a^
IDF	0.62 ± 0.01 ^b^	4.13 ± 0.02 ^a^	0.80 ± 0.01 ^b^	4.11 ± 0.22 ^a^

^1^ Means ± standard deviation. Lower case letters in the same column denote significant differences (*p* < 0.05) among formulations. All parameters are expressed as g/100 g of dry weight, except for moisture content. HMWDF, high molecular weight dietary fiber; HMWSDF, high molecular weight soluble dietary fiber; IDF, insoluble dietary fiber; ^2^ Frozen snack formulations are according to Table 1.

**Table 4:** Evaluation ^1^ of total phenolic content (TPC), total monomeric anthocyanin content (TAC), and antioxidant capacity (ABTS and DPPH) of frozen fermented dairy snack formulations ^2^ before and after in vitro digestion.

	TPC	TAC	ABTS	DPPH
	(mg GAE/g d.w.)	(µg C3G/g d.w.)	(mg Trolox/g d.w.)	(mg Trolox/g d.w.)
Before digestion				
LA5	1.04 ± 0.08 ^b^	-	1.11 ± 0.12 ^b^	1.96 ± 0.04 ^a^
LA5-BERRY	1.74 ± 0.03 ^a^	42.92 ± 0.88 ^a^	4.09 ± 0.12 ^a^	1.83 ± 0.16 ^a^
LGG	0.95 ± 0.08 ^b^	-	0.90 ± 0.01 ^b^	1.90 ± 0.26 ^a^
LGG-BERRY	1.85 ± 0.09 ^a^	46.57 ± 1.31 ^a^	4.14 ± 0.18 ^a^	1.92 ± 0.10 ^a^
After digestion				
LA5	5.19 ± 0.17 ^c^*	-	14.80 ± 0.70 ^b^*	22.87 ± 1.07 ^a^*
LA5-BERRY	8.74 ± 0.23 ^a^*	14.66 ± 2.16 ^a^*	20.05 ± 0.22 ^a^*	23.97 ± 1.63 ^a^*
LGG	4.98 ± 0.33 ^c^*	-	13.66 ± 0.32 ^b^*	24.56 ± 1.36 ^a^*
LGG-BERRY	7.62 ± 0.11 ^b^*	15.11 ± 0.10 ^a^*	19.84 ± 0.37 ^a^*	24.69 ± 1.21 ^a^*

ABTS, 2,2-azino bis (3-ethylbenzo thiazoline 6 sulfonic acid); DPPH, (2,2-difenil-1-picrilhidrazil); GAE, gallic acid equivalent; C3G, cyanidin-3-glucoside; d.w., dry weight; ^1^ Means ± standard deviation. Lower case letters in each column indicate significant differences (*p* < 0.05) among formulations. Asterisks indicate significant differences in the formulations (*p* < 0.05) before and after in vitro digestion; ^2^ Frozen snack formulations are described in Table 1.

## Data Availability

The original contributions presented in the study are included in the article, further inquiries can be directed to the corresponding author.
